# Is there a connection between spine alignment, chest mobility, shoulder joint and respiratory parameters of patients with ankylosing spondylitis?

**DOI:** 10.1007/s00296-024-05642-0

**Published:** 2024-06-25

**Authors:** Daniel Szewczyk, Teresa Sadura-Sieklucka, Beata Tarnacka, Beata Sokołowska

**Affiliations:** 1 Department of Rehabilitation, National Institute of Geriatrics, Rheumatology and Rehabilitation, Warsaw, Poland; 2 Department of Geriatrics, National Institute of Geriatrics, Rheumatology and Rehabilitation, Warsaw, Poland; 3 Medical University of Warsaw, Warsaw, Poland; 4 Mossakowski Medical Research Centre, Polish Academy of Sciences, Warsaw, Poland

**Keywords:** Ankylosing spondylitis, Respiratory parameters, Chest mobility, Spine

## Abstract

**Introduction:**

Ankylosing spondylitis is chronic progressive disease, which decrease functions of musculoskeletal system including chest area. Those changes influences respiratory mechanics, worsen conditions of proper ventilation of lungs.

**Objectives:**

Rating of functional and respiratory parameters and dependence between them at patients with ankylosing spondylitis.

**Materials & methods:**

The study included 45 patients with diagnosed ankylosing spondylitis. Chest and upper limbs mobility, resting spinal curvature alignment were assessed, and respiratory parameters were measured in a plethysmographic chamber JAGGER MasterScreen Body.

**Results:**

Ankylosing spondylitis patients had lower respiratory parameters especially sReff, and FRC. Restriction of chest and upper limbs mobility was also demonstrated. Forward head extension was observed based on the occipital wall test. Correlations between functional parameters and correlations between functional and respiratory parameters were shown, in particular MIP, MEP, sReff, Rtot, TLC, ERV.

**Conclusions:**

The study confirmed a decrease in functional and respiratory parameters in the examined patients with ankylosing spondylitis compared to the applicable standards. A significant relationship was found between functional parameters in the upper body and respiratory parameters, which worsen with increasing thoracic dysfunction. The obtained results indicate the directions of therapy that should be taken into account to improve respiratory parameters and reduce respiratory dysfunction in these patients. Chest-focused physiotherapy appears to be an important element in improving function in patients with ankylosing spondylitis.

## Introduction

Ankylosing spondylitis (AS) is a progressive autoimmune disease that significantly and gradually impairs a patient’s functional capacity. As a result of the ongoing inflammatory process, the spine stiffens and its curvature changes, which affects the patient’s posture. The sacrum becomes vertically aligned, and there is a shallowing of the lumbar lordosis, thoracic hyperkyphosis, and head protraction due to the kyphotic alignment of the cervical spine [1, 2]. Patients with ankylosing spondylitis experience abnormal breathing patterns, which in turn adversely affects the results of respiratory tests [3]. Due to changes in the curvature of the spine, the entire chest and trunk work under new and inferior conditions [4, 5]. Some muscles shorten and others weaken, with the consequence that most respiratory muscles work abnormally [6, 7]. As a result of the stiffening of the spine and intertransverse joints, there is reduced mobility and flexibility of the entire chest, especially the ribs [8]. Dysfunction of the shoulder girdle is also frequently observed, which is associated with abnormal thoracic alignment and dysfunction of the trunk muscles [9–11].

The study aimed to evaluate functional and respiratory parameters in patients with ankylosing spondylitis, and to determine the relationships between selected functional and respiratory parameters in these patients.

## Patients and methods

Forty-five patients participated in the study. Patients aged 18–75 years diagnosed with AS who agreed to participate in the study were enrolled. The exclusion criteria encompassed respiratory and cardiovascular diseases, cardiac surgery, damage to the musculoskeletal system in the upper limbs (bone fractures, sprains and dislocations of joints), chest (broken ribs, sternum, clavicle), and/or other chronic diseases of the skeletal-articular system limiting mobility of the joints in the upper limbs. Due to the fact that each patient had mobility limitations in the examined areas, it was not possible to assess whether they resulted from the primary disease or injury/surgery. Therefore, post-trauma/surgery patients were excluded from the study. In each patient, chest expansion was measured at the level of the fourth intercostal space and at the level of the shaft of the tenth thoracic vertebra using a centimeter tape. In addition, the angular alignment of the curvature of the spine was measured using a Baseline 12-1056 inclinometer and the mobility of the shoulder joint was assessed using a goniometer.

A plethysmographic study was also performed in a JAGGER MasterScreen Body plethysmographic chamber. The study evaluated the following parameters sReff (*effective specific airway resistance*), Rtot (*total specific airway resistance*), FRC (*functional residual capacity*), ERV (*expiratory reserve volume*), RV (*residual volume*), TLC (*total lung capacity*), Rv%TLC, VC (*vital capacity*), MIP (*maximum inspiratory pressure*), and MEP (*maximum expiratory pressure*). Since respiratory values are individually matched to patients according to their gender, height, weight, and age to standardize values, data were expressed as a percentage of values achieved to normative values. The research project was approved by the Bioethics Committee at NIGRiR, No. KBT-1/06/2019. Each participant gave written consent to participate in the study. Also, each participant was provided with information about the study, as well as the right to withdraw from the study at any time.

### Statistical analysis

Statistical analysis was performed using STATISTICA 10.0 PL software. Values were presented as means and standard deviations. The distribution of the variables assessed by the Shapiro-Wilk test showed a non-Gaussian distribution. The non-parametric Wilcoxon test was used to assess statistical significance. Correlations between parameters were analyzed using Spearman’s rank correlation coefficient. A p-value less than 0.05 was considered statistically significant.

## Results

Forty-five patients diagnosed with ankylosing spondylitis according to the New York criteria were included in the study [12]. There were 31 men and 14 women in the study group, the average age of the subjects was 46.4 ± 12.9 (median- 43, min- 19, max- 73) years, and the average disease duration was 14.4 ± 11.6 (median- 10.5, min- 0.5, max- 48) years. The results of the study showed that in patients with ankylosing spondylitis, the values of respiratory parameters (MIP, MEP, sReff, sRtot, FRCpl, ERV, RV, TLC, VC) were reduced compared to normative values for healthy subjects (Table 1). The largest difference was observed in the FRC and sReff parameters. The patients were also characterized by limited shoulder joint mobility, limited chest expansion and head protrusion in the occiput-wall test, as shown in Table 2. The relationship between functional parameters and characteristics including age, disease duration and spinal angulation was also assessed (Table 3). Many correlations were demonstrated between functional parameters (age, disease duration, spine position, spine mobility, upper limb mobility) both among, and between respiratory parameters. The tests showed the progressive nature of the disease and the deteriorating posture of the patients due to the correlation between patient age, duration of the disease and the OWD test and the position of the cervical spine (Table 3). The positioning of the lumbar spine showed a relationship only with the thoracic spine. The thoracic spine influenced the upper limbs and the OWD test. The cervical spine had the greatest impact on OWD. When it comes to the influence of functional parameters on respiratory parameters, they are numerous (Table 4), however, the greatest influence seems to be on the TLC parameter due to the largest number of correlations to functional parameters. The VC parameter was also characterized by a large number of correlations. As for the functional parameters, the resting position of the thoracic spine exhibits the highest number of correlations with respiratory parameters.

**Table 1:** Values of measured respiratory parameters in patients with ankylosing spondylitis

Parameters	Mean ± SD
MIP (median; min-max)	80 ± 29% (75%; 19–142%)
MEP (median; min-max)	81 ± 31% (76%; 21–170%)
sReff (median; min-max)	61 ± 21% (57%; 22–105%)
sRtot (median; min-max)	80 ± 28% (73%; 26–144%)
FRC (median; min-max)	58 ± 24% (103%; 53–205%)
ERV (median; min-max)	111 ± 28% (136%; 41–235%)
RV (median; min-max)	138 ± 47% (82%; 18–242%)
TLC (median; min-max)	86 ± 16% (89%; 37–129%)
Rv%TLC (median; min-max)	97 ± 38% (93%; 22–200%)
VC (median; min-max)	85 ± 20% (87%; 38–128%)

MIP- *maximal inspiratory pressure*, MEP- *maximal expiratory pressure*, sRAW- *specific airway resistance*, sRtot- total sRaw (*total specific airway resistance*), sReff- effective sRaw (*effective specific airway resistance*), FRC- *functional residual capacity*, ERV- *expiratory reserve volume*, RV- *residual volume*, TLC- *total lung capacity*, VC- *vital capacity*

**Table 2:** Values of selected measurements in patients with ankylosing spondylitis

Parameters	Mean ± SD (median; min-max)
Occiput to wall distance(cm) (OWD)	9 ± 17 (2; 0–51)
Right shoulder joint flexion (°) (RSF)	151 ± 21 (155; 83–176)
Right shoulder joint abduction (°) (RSA)	148 ± 21 (150; 89–179)
Left shoulder joint flexion (°) (LSF)	151 ± 24 (157; 70–180)
Left shoulder joint abduction (°) (LSA)	147 ± 29 (151; 76–180)
Chest expansion at the level of the fourth intercostal space (cm) (Th4)	4.0 ± 1.6 (4; 1.5-8)
Chest expansion at the level of the shaft of the tenth thoracic vertebra (cm) (Th10)	4.7 ± 1.7 (5; 2–9)

OWD- *occiput to wall distance*, Th4- *chest expansion at the level of the fourth intercostal space*, Th10- *chest expansion at the level of the shaft of the tenth thoracic vertebra*, RSF- *right shoulder joint flexion*, RSA- *right shoulder joint abduction*, LSF- *left shoulder joint flexion*, LSA- *left shoulder joint abduction*

**Table 3:** Values of correlation coefficients of functional parameters with age and disease duration

Parameters	Age	Disease duration	L-S	Th	C
OWD	0.41*	0.47*	-0.22	-0.52*	0.55*
TH4	-0.14	-0.12	-0.05	0.20	-0.09
TH10	-0.30*	-0.19	-0.13	-0.19	-0.22
RSF	-0.32*	-0.36*	0.11	0.25	-0.55*
RSA	-0.28	-0.32*	-0.02	0.25	-0.25
LSF	-0.12	-0.30*	0.07	0.36*	-0.29
LSA	-0.01	-0.23	0.04	0.36*	-0.12
L-S	-0.27	0.09	1.00	0.61*	-0.17
Th	-0.06	-0.18	0.61*	1.00	-0.06
C	0.44*	0.34*	-0.17	-0.06	1.00

OWD- *occiput to wall distance*, Th4- *chest expansion at the level of the fourth intercostal space*, Th10- *chest expansion at the level of the shaft of the tenth thoracic vertebra*, RSF- *right shoulder joint flexion*, RSA- *right shoulder joint abduction*, LSF- *left shoulder joint flexion*, LSA- *left shoulder joint abduction*, LS- *lumbar spine alignment*, TH- *thoracic spine alignmen*t, C- *cervical spine alignment*

**Table 4:** Values of correlation coefficients of functional and respiratory parameters and with age and disease duration

Parameters	MIP	MEP	sReff	Rtot	Reff	FRC	ERV	RV	TLC	Rv%TLC	VCin
Age	0.17	0.16	0.28	0.31*	0.28	0.12	0.34*	-0.11	-0.02	-0.10	0.15
Disease duration	-0.11	-0.01	-0.18	-0.14	-0.13	0.19	0.27	-0.00	-0.17	0.03	0.03
OWD	-0.11	-0.11	-0.19	-0.19	-0.10	0.06	0.25	-0.05	-0.37*	0.02	-0.20
Th4	0.12	0.26	0.20	0.07	0.00	0.21	0.21	0.09	0.55*	-0.11	0.51*
Th10	0.32*	0.35*	0.16	0.22	0.07	0.18	0.26	-0.02	0.27	-0.18	0.40*
LS	0.07	0.17	0.15	0.06	0.08	-0.01	-0.18	0.23	0.3*	0.18	0.11
TH	0.23	0.32*	0.38*	0.32*	0.25	0.21	-0.04	0.14	0.35*	0.17	0.13
C	-0.01	-0.03	0.14	0.16	0.26	0.10	0.16	0.10	-0.18	0.21	-0.13
RSF	0.21	0.10	0.20	0.12	0.05	0.13	0.04	0.15	0.42*	-0.01	0.33*
RSA	0.17	0.15	0.21	0.05	-0.00	0.18	0.02	0.23	0.42*	0.10	0.22
LSF	0.33*	0.34*	0.39*	0.29	0.24	0.02	-0.01	0.06	0.39*	-0.04	0.30*
LSA	0.33*	0.35*	0.27	0.18	0.10	0.09	0.09	0.12	0.29	0.04	0.15

OWD - Occiput to Wall Distance, Th4- chest expansion at the level of the fourth intercostal space, Th10- chest expansion at the level of the shaft of the tenth thoracic vertebra, RSF- right shoulder joint flexion, RSA- right shoulder joint abduction, LSF- left shoulder joint flexion, LSA- left shoulder joint abduction, Th4- chest expansion at the level of the fourth intercostal space, Th10- chest expansion at the level of the shaft of the tenth thoracic vertebra, LS- lumbar spine alignment, TH- Thoracic spine alignment, C- cervical spine alignment. MIP- Maximal inspiratory pressure, MEP- maximal expiratory pressure, sRAW- specific airway resistance, sRtot- total sRaw(total specific airway resistance, sReff- effective sRaw(effective specific airway resistance), FRC- functional residual capacity, ERV- Expiratory reserve volume, RV- residual volume, TLC- total lung capacity, VC- vital capacity

## Discussion

In the research context, a debate is developing about the relationship between spinal alignment and respiratory parameters. Numerous studies have focused on analyzing the influence of spinal alignment on respiratory function. While reports suggest that the two body spheres are interrelated, there is also much controversy and differences in interpreting study results. Studies on how spinal alignment affects respiratory parameters shed light on the complex relationship between thoracic biomechanics and respiratory function. In patients with ankylosing spondylitis, lower respiratory parameters were observed compared to healthy subjects. In terms of parameters (TLC, RAW, VC, PFT, FVC, MIP, MEP) [13–15]. Similar results were observed in our study. Patients with ankylosing spondylitis are characterized by reduced inspiratory and expiratory muscle strength [3]. Disturbed breathing patterns, decreased respiratory parameters, and reduced muscle strength significantly affect their respiratory capacity [16]. Osteoarticular dysfunctions leading to abnormal body alignment contribute to abnormal breathing patterns, as well as impaired respiratory function. Our study showed correlations between these two phenomena. Restriction of upper limb mobility, expansion of the thorax, and large distance of the occiput from the wall were observed. The study found correlations between the patient’s age and the head-to-wall distance in the occiput to-wall test. This result confirms the progressive nature of the disease, as well as its significant impact on patient posture. Similar results were also observed in the study by Maksymowych et al. [17]. However, no correlation was observed between chest expansion and patient age. A study by the Wirth group also found a correlation between thoracic expansion and head protraction [16]. Resting head position correlates strongly with the occipital-wall test in patients with ankylosing spondylitis. This test depends on the duration of the disease, as well as the age of the patient. Our study showed no statistically significant correlation between patient age and upper limb mobility. Maksymowych et al. [17] also confirm the results obtained. A statistically significant negative correlation was observed between upper limb mobility and disease duration, indicating a progressive, disabling course of the disease [17]. There is a correlation between the angular position of the thoracic spine and the mobility of the upper extremities [18–21]. The angular position of the cervical spine also affects the mobility of the upper limb [22]. Similar results were shown in the present study. The occiput-wall test also depends on the shape of the chest and its alignment [23]. Functionally, the spine constitutes a single unit. The alignment of the articular surfaces of the segments with respect to each other forces the alignment of the adjacent segments so that the alignment of the lumbar spine affects the alignment of the thoracic spine [24]. This relationship is inversely proportional to a decrease in lumbar lordosis and contributes to a deepening of thoracic kyphosis [25]. In both the literature [25] and our study, no correlation was observed between thoracic and lumbar spine alignment and the cervical spine. However, other studies [26, 27] have observed such a relationship. A correlation was found between chest stiffness, chest expansion and measurements of respiratory parameters, including respiratory muscle strength [3]. The lumbar spine has also been shown to correlate with respiratory parameters, which may suggest an important role of the diaphragm in these patients [28]. Not only the lumbar spine but also chest expansion, cervical spine mobility and chest mobility have been shown to correlate with respiratory parameters [29]. However, no such relationships were observed in our study. Berdal et al. [4] described correlations between spinal mobility and respiratory parameters. The authors also observed a correlation between the occiput-wall test and lung function [4]. A negative correlation was noted between disease duration and TLC, VC parameters [13, 17]. This relationship was not observed in our study. TLC, VC parameters correlated with thoracic expansion [13]. A similar relationship was observed in our study, TLC (*r* = 0.55 vs. *r* = 0.55), VC (*r* = 0.58 vs. *r* = 0.51). Also, respiratory muscle strength (MIP, MEP) correlates with chest expansion [2]. Our study confirmed this relationship, but only at the Th10 level. A study by Culham et al. found a negative correlation between thoracic shape and thoracic expansion and TLC and VC parameters in women with osteoporosis [30]. These patients also showed reduced chest expansion, as well as changes in spinal curvature, which may speak to postural change and its respiratory correlates, as in patients with ankylosing spondylitis. Our research confirms this association in patients with ankylosing spondylitis. In contrast, studies on older women did not confirm these results [31]. Instead, they showed a correlation between lumbar spine alignment and respiratory parameters, which our study did not identify. This study also found a correlation between patient age and the ERV parameter, and between the sReff and Rtot coefficients with upper limb mobility and thoracic spine alignment. Based on imaging studies, Sadura-Sieklucka et al. [32] showed how rehabilitation in the chest area had a positive impact on the improvement of its functioning, which resulted in improved respiratory parameters. Szewczyk et al. [33] showed that rehabilitation aimed at improving motor functions had a positive effect on respiratory parameters in patients with AS.

## Conclusion

The study showed that AS patients had reduced functional and respiratory parameters. The results reveal a range of mutual correlations. The study findings suggest that at least some of the respiratory problems in AS patients are correlated with dysfunctions of the musculoskeletal system in the thoracic region (the largest number of correlations were found there). These results open a new path for physiotherapy in these patients, which should be strongly concentrated on these body areas. In addition to breathing and aerobic exercises, it may be beneficial to provide this group of patients with stretching exercises that improve the range of motion and posture, and with manual therapy.

### Limitations of the study

The study included only 45 participants. The group was estimated based on the literature, but it was not calculated with statistical power what group is required for certain results. The study group was not large, but it nevertheless showed a certain pattern of dysfunction among the patients. The research should be expanded to include a larger number of participants. The study group could be narrowed to be more uniform in terms of age and disease duration, while excluding the aspect of loss of range of motion related to the aging process. The study did not take into account the physical activity of the patients, which made some of them more upright (as demonstrated in the maximum plethysmography values), while some patients were less physically active. Due to the inability to perform blood tests in the ward, the current severity of the disease in patients was not taken into account, which may affect the range of motion of the upper limb and chest.
